# The impact of blade geometry (nonflat, flat or hybrid) and metallurgical composition on the mechanical performance of NiTi endodontic instruments: A multimethod laboratory study

**DOI:** 10.1111/iej.14249

**Published:** 2025-05-03

**Authors:** Emmanuel J. N. L. Silva, Jorge N. R. Martins, Thyago Oliveira Cardoso, Mylena do Rosário Pereira, Murilo Priori Alcalde, Victor T. L. Vieira, Abayomi O. Baruwa, Francisco Manuel Braz Fernandes, Marco A. Versiani

**Affiliations:** ^1^ School of Dentistry Grande Rio University (UNIGRANRIO) Rio de Janeiro Brazil; ^2^ Department of Endodontics Rio de Janeiro State University Rio de Janeiro Brazil; ^3^ Deparment of Endodontics Fluminense Federal University Niterói Brazil; ^4^ Faculdade de Medicina Dentária Universidade de Lisboa Lisbon Portugal; ^5^ Grupo de Investigação Em Bioquimica e Biologia Oral, Unidade de Investigação Em Ciências Orais e Biomédicas (UICOB), Faculdade de Medicina Dentária Universidade de Lisboa Lisbon Portugal; ^6^ Centro de Estudo de Medicina Dentária Baseada na Evidência (CEMDBE) – Cochrane Portugal, Faculdade de Medicina Dentária Universidade de Lisboa Lisbon Portugal; ^7^ LIBPhys‐FCT UID/FIS/04559/2013, Faculdade de Medicina Dentária Universidade de Lisboa Lisbon Portugal; ^8^ Department of Endodontics, Bauru Dental School São Paulo University Bauru Brazil; ^9^ Department of Clinical Sciences, College of Dentistry Ajman University Ajman United Arab Emirates; ^10^ CENIMAT/I3N, Department of Materials Science, NOVA School of Science and Technology Universidade NOVA de Lisboa Caparica Portugal; ^11^ Dental Specialty Center Brazilian Military Police Belo Horizonte Minas Gerais Brazil

**Keywords:** differential scanning calorimetry, energy‐dispersive X‐ray spectroscopy, endodontics, flat‐side instruments, mechanical performance, metallurgical features

## Abstract

**Aim:**

To evaluate the influence of blade design (conventional, flat and hybrid) and metallurgical properties on the mechanical performance of nickel‐titanium endodontic instruments.

**Methodology:**

Two hundred and seven NiTi instruments (25 mm in length) with three different blade designs were selected for analysis: conventional (*n* = 69, CC One Blue, size 25/0.08v), flat (*n* = 69, Platinum V.EU, size 25/0.06) and hybrid (*n* = 69, Flash Endo Power, size 25/0.06v). The instruments were evaluated regarding geometric design (scanning electron microscopy), alloy elements composition (energy‐dispersive X‐ray spectroscopy) and phase transformation temperatures (differential scanning calorimetry). Additionally, their mechanical behaviour was investigated by testing cyclic fatigue resistance, torsional resistance, bending resistance, buckling resistance, cutting efficiency and microhardness. Statistical significance was determined using One‐Way anova and Kruskal–Wallis tests (*α* = 5%).

**Results:**

Platinum V.EU and Flash instruments exhibited design inconsistencies within the same lot, including nonstandard positioning and variations in the length of the flat side. All instruments were composed of a nickel‐titanium alloy with equiatomic ratios of nickel and titanium. At 20°C, Flash instruments exhibited a mixed R‐phase and austenitic arrangement, transitioning fully to austenitic at 36°C, while CC One Blue and Platinum V.EU displayed a complete R‐phase at 20°C and retained a mixed R‐phase and austenitic arrangement at 36°C. The CC One Blue exhibited superior performance in time to fracture (156 ± 34 s), maximum torque (1.5 N·cm) and buckling strength (372 ± 31 gf) (*p* < .0001), while no differences were found in maximum rotation angle (*p* = .602). In terms of flexibility, the Flash (328 gf) and CC One Blue (341 gf) outperformed the Platinum V.EU (376 gf) (*p* = .006). Flash (121 gf) and CC One Blue (137 gf) also outperformed Platinum V.EU (253 gf) in terms of cutting efficiency (*p* < .0001). Conversely, the Platinum V.EU demonstrated significantly higher microhardness (386 ± 45 HVN) compared to CC One Blue and Flash (*p* = .0340).

**Conclusions:**

Overall, instruments featuring either flat‐side (Platinum V.EU) or hybrid (Flash) active blades demonstrated inferior mechanical performance compared to the conventional nonflat instrument (CC One Blue).

## INTRODUCTION

The introduction of nickel‐titanium (NiTi) instruments has revolutionized endodontics, particularly in root canal shaping procedures. Advances in metallurgy and instrument design have yielded innovations characterized by variations in kinematics, surface treatment and cross‐sectional geometry, significantly enhancing procedural efficiency and reducing the risk of iatrogenic errors such as canal transportation and ledge formation (Martins et al., 2022; Silva et al., 2020). These advancements have not only improved the technical precision of root canal treatments but have also contributed to better clinical outcomes, such as reduced treatment times and increased success rates (Bürklein & Arias, 2022). Despite these improvements, the inherent challenges associated with NiTi instruments—such as deformation and fracture under clinical conditions—remain key concerns for clinicians (McGuigan et al., 2013; Ng et al., 2011).

To overcome some of these challenges, the Tango‐Endo system (Essential Dental Systems, Hackensack, USA) was introduced in 2015, representing the first commercial application of the flat‐side concept in endodontic instrument design (Essential Dental Systems, 2015). The underlying rationale for this design lies in the hypothesis that incorporating a longitudinal flat along the blade could reduce the metallic mass, thereby enhancing the instrument's flexibility, improving resistance to cyclic fatigue and facilitating more efficient debris removal during canal instrumentation. In subsequent years, this modified blade geometry was adopted by other manufacturers, seeking to further optimize the instrument's performance. By reducing blade engagement with canal walls and directing debris more effectively from the cutting flutes to the flat‐side relieving area, these designs were intended to extend the instrument's fatigue lifespan, streamline debris transportation and ultimately improve cleaning efficiency within the root canal system (Bondent, 2019; Fanta Dental, 2023; Mighty Medico, 2023; United Dental Group, 2024). Despite these theoretical benefits, the actual performance of flat‐side instruments has largely failed to meet expectations. Some studies have highlighted significant limitations, including reduced cyclic fatigue resistance, inconsistencies in manufacturing quality, such as irregular flat‐side geometry and heat treatment issues (Martins et al., 2023; Ubaed & Bakr, 2022), and high‐stress distribution patterns on canal walls (Carvalho et al., 2025). A comprehensive multimethod study—focused on noncommercial comparisons between flat and nonflat instruments—clearly demonstrated that the theoretical advantages of flat‐side systems do not materialize in practice. The study concluded that the creation of a flat‐side instrument does not yield any substantial advantages over conventional designs in terms of mechanical performance, challenging the claims of manufacturers and underscoring the need for further innovation and refinement in instrument design (Silva et al., 2023).

Building on the concept of flat‐side designs, a hybrid approach has recently been introduced, combining features of both flat and nonflat geometries. The Flash Endo Power instrument (Bondent, Shangai, China) features a conventional cross‐sectional design at its tip (approximately 6 mm in length) while the remainder of the active blade is abraded to form a flat surface. According to the manufacturer, this design aims to merge the flexibility and debris‐removal efficiency of flat‐side instruments with the cutting effectiveness and structural stability associated with conventional NiTi instruments (Bondent, 2025). Although previous studies have highlighted significant limitations (Martins et al., 2023; Silva et al., 2023) and reported mixed findings regarding the performance of flat‐side designs (Di Nardo et al., 2020; Gambarini et al., 2019), a comprehensive multimethod comparison of hybrid instruments with those of similar tip size and original cross‐sectional geometry (S‐shape) but differing blade designs could provide valuable insights into the influence of design features on the mechanical behaviour of endodontic instruments. Therefore, this study employed a comprehensive multimethod approach to investigate the metallurgical properties and mechanical performance of three heat‐treated NiTi instruments, produced by the same manufacturer (Bondent, Shangai, China) and categorized into conventional (CC One Blue), flat‐side (Platinum V.EU) and hybrid (Flash Endo Power) blade designs. The instruments were analysed based on three key aspects: design, assessed using scanning electron microscopy (SEM); metallurgy, evaluated through energy‐dispersive X‐ray spectroscopy (EDS) and differential scanning calorimetry (DSC); and mechanical performance, which included tests for cyclic fatigue resistance, torsional resistance, bending resistance, buckling resistance, cutting efficiency and microhardness. By comparing instruments with these varying blade geometries under controlled conditions, the study sought to elucidate the influence of design features on mechanical behaviour. The null hypothesis states that differences in blade design would not significantly affect the mechanical performance of the tested instruments.

## MATERIALS AND METHODS

This manuscript complies with the Preferred Reporting Items for Laboratory studies in Endodontology (PRILE) guidelines (Nagendrababu et al., 2021) (Figure S1) and the respective checklist set forth by the International Endodontic Journal.

### Sample selection

**FIGURE 1:**
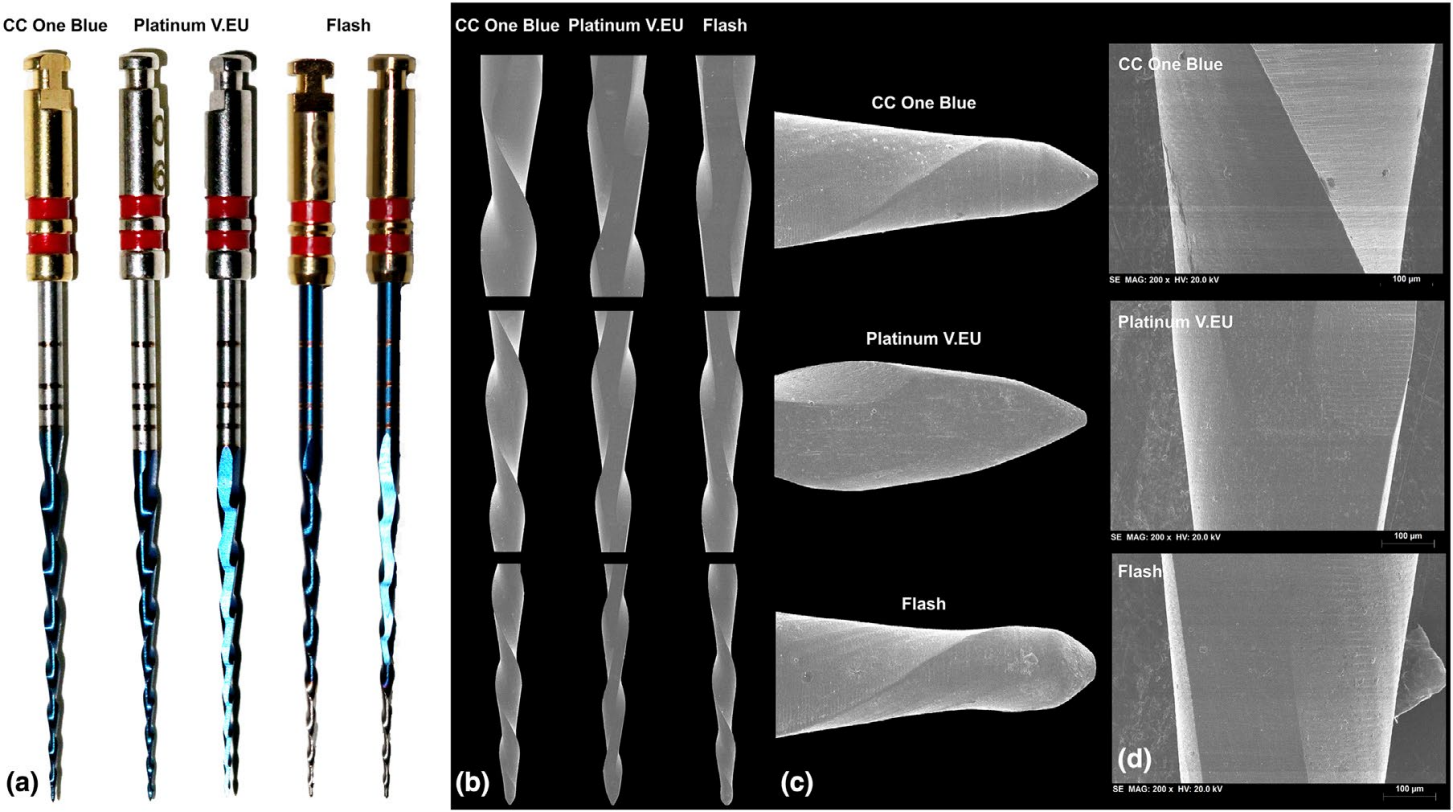
Representative images of the assessed instruments. (a) The macro photographs display the distinctive blade designs, with the Platinum V.EU instrument characterized by a full‐length flat side and the Flash instrument featuring a hybrid design with a blade presenting flat and nonflat areas. The Flash instrument also presents two different alloy surface colours, blue and white, which are clearly visible (left). (b) The SEM images focus on the blade designs (centre‐left) and highlight the variations in (c) tip geometries across all instruments, with none exhibiting an active tip design. (d) Additionally, the SEM images reveal that the surface finishing is marked by smooth, parallel manufacturing lines, with minor irregularities noted (centre‐right and right).

This study compared the geometric design, metallurgical properties and mechanical performance of 207 NiTi instruments (25 mm in length) produced by the same manufacturer (Bondent, Shangai, China) and categorized into three groups based on their blade design: conventional (*n* = 69, CC One Blue, size 25/0.08v, Lot 1042024), flat (*n* = 69, Platinum V.EU, size 25/0.06, Lot 202108300001) and hybrid (*n* = 69, Flash Endo Power, size 25/0.06v, Lot 202311280001) (Figure 1). All instruments underwent microscopic inspection at 13.6× magnification with LED illumination (Opmi Pico; Carl Zeiss Surgical, Jena, Germany) prior to testing to detect significant defects, such as blade design irregularities or unwinding, and, as no defects were found, all instruments were considered suitable for inclusion.

### Design

Six instruments from each group were randomly selected for examination under a dental microscope (Opmi Pico, Carl Zeiss Surgical, Jena, Germany) at 13.6× magnification, with images captured using a digital camera (Canon EOS 500D; Canon, Tokyo, Japan) to assess active cutting blade length, spiral count, spirals per millimetre, spiral direction and manufacturing inconsistencies. These instruments were subsequently mounted on a file holder and analysed using a scanning electron microscope (SEM) (S‐2400, Hitachi, Tokyo, Japan) to evaluate their spiral geometry (symmetrical or asymmetrical), tip design (active or nonactive), surface finishing marks from the manufacturing process, and any minor defects or irregularities resulting from manufacturing.

### Metallurgy

A semi‐quantitative elemental analysis was performed on three instruments from each group using energy‐dispersive X‐ray spectroscopy (EDS). The first instrument was used to obtain the results, while the other two instruments were tested to verify the reproducibility and consistency of the findings. The analysis was conducted with a standard SEM unit (DSM‐962, Carl Zeiss Microscopy GmbH, Jena, Germany) equipped with an Inca X‐act EDS detector (Oxford Instruments NanoAnalysis, Abingdon, UK). The system operated at 20 kV and 3.1 amps, collecting data from a 500 μm × 500 μm area over a 1‐min period at a 25 mm working distance, following a 10‐min vacuum process. The atomic number (Z), absorption (A) and fluorescence (F) correction method (ZAF) was applied. The corrected data were processed using Microanalysis Suite v.4.14 (Oxford Instruments NanoAnalysis, Abingdon, UK) to determine the proportions of metallic elements in the samples. Differential scanning calorimetry (DSC) tests were conducted following ASTM International guidelines (ASTM International, 2004) to assess phase transformation temperatures, ensuring reliable measurement of thermal events, including martensitic and austenitic transitions, by monitoring heat flow changes with temperature. Small fragments (4–5 mm, 5–10 mg) were cut from each reference instrument's active blade, etched in a solution of 45% nitric acid, 25% hydrofluoric acid and 30% distilled water for 2 min, neutralized with distilled water, and placed in an aluminium pan inside the DSC device, with an empty pan as the control. The heat cycle, lasting 1 h and 40 min under a nitrogen gas atmosphere, ranged from −150°C to 150°C with a 10°C per minute increase, and the resulting DSC data and graphs were processed using Netzsch Proteus Thermal Analysis software (Netzsch‐Gerätebau GmbH, Selb, Germany). For the Flash Endo Power instrument, both the flat and nonflat surfaces were analysed using EDS and DSC tests.

### Mechanical performance

Seven parameters were evaluated to assess the mechanical performance of the instruments: time to fracture, maximum torque, maximum rotation angle, bending strength, buckling strength, cutting efficiency and microhardness. To determine the ideal sample size for these tests, a calculation was performed based on the most variable results from an initial set of six tests. The sample size calculation accounted for an alpha‐type error of 0.05, a power of 80% and effect sizes (± standard deviation) as follows: 99.67 (± 56.90) for time to fracture (CC One Blue vs. Platinum V.EU), 0.47 (± 0.31) for maximum torque (CC One Blue vs. Platinum V.EU), 25.83 (± 69.28) for maximum rotation angle (Platinum V.EU vs. Flash), 68.25 (± 46.81) for bending strength (Platinum V.EU vs. Flash), 172.50 (± 89.75) for buckling strength (CC One Blue vs. Flash) and 133.17 (± 67.63) for cutting efficiency (Platinum V.EU vs. Flash). The calculated required sample sizes were 7, 8, 113, 9, 6 and 6, respectively. Given the exceptionally large sample sizes required for the maximum rotation angle parameter, which would likely result in statistically significant differences that have minimal practical or clinical relevance, the sample size calculation was adjusted to focus on the outcomes of the other parameters. Consequently, the final sample size was determined to be 10 instruments, ensuring a balance between statistical power and the practical significance of the results. For microhardness, the first five indentations from one instrument were considered. Based on an effect size of 65.17 (± 59.85) (CC One Blue vs. Platinum V.EU), the required sample size was determined to be 15 indentations.

The cyclic fatigue test was performed at room temperature (20 ± 1°C) with instruments mounted on a 6:1 reduction handpiece (Sirona Dental Systems GmbH, Bensheim, Germany) powered by a torque‐controlled motor (AI‐Motor; Woodpecker, Guilin, China) set at 350 RPM and 2.4 N.cm torque. The counterclockwise and clockwise angles were 150° and 30° for CC One Blue and Flash instruments, and 30° and 150° for Platinum V.EU instruments. Although the Platinum V.EU is designed for use in continuous rotation, it was tested in reciprocating motion, with its movement reversed due to its oppositely oriented spiral flutes, to match the kinematics of the other instruments and ensure a standardized comparison. The instruments were operated in a static position within a nontapered stainless‐steel curved tube apparatus with 19 mm length, 1.3 mm internal diameter and with a 6 mm radius and 86° angle, with the maximum stress area being applied at the 9‐mm level (D9) of the instrument's length. Fracture was determined through visual and auditory observation, and the time to fracture (in seconds) was recorded using a digital chronometer.

Torsional strength was assessed by measuring maximum torque (N·cm) and maximum angle of rotation (°) following the international standard ISO 3630‐3631 (ISO 3630‐3631, 2008). In this test, the instruments were positioned straight and secured in a torsiometer (TT100; Odeme Dental Research, Luzerna, Brazil), with the apical 3 mm firmly clamped. They were then rotated at a constant speed of 2 rpm until fracture, following the specific kinematics of each instrument: clockwise for Platinum V.EU and counterclockwise for CC One Blue and Flash. The maximum torque sustained before rupture (measured in N·cm) and the angle of rotation (in degrees) were recorded using dedicated software (Odeme Analysis TT100, Odeme Dental Research, Luzerna, Brazil).

Bending resistance was assessed by measuring the maximum bending load (gf) in accordance with the international standard ISO 3630‐1 (ISO 3630‐3631, 2008). For this test, the instruments were secured in a file holder and positioned at a 45° angle to the floor, while their apical 3 mm were attached to a wire connected to a universal testing machine (DL‐200 MF; EMIC, São José dos Pinhais, Brazil). A 20 N load was applied at a constant speed of 15 mm/min until the instrument achieved a 45° displacement. The maximum load required to reach this displacement was recorded in gram‐force (gf).

Buckling resistance was assessed using a universal testing machine (DL‐200 MF; EMIC, São José dos Pinhais, Brazil) equipped with a 1 kN load cell. Each instrument was positioned vertically, with its handle securely fixed to the machine head and its tip inserted into a small slot on a stainless‐steel test base to ensure stability (Lopes et al., 2012) A compressive force was then applied along the instrument's longitudinal axis at a constant speed of 1 mm/min, moving from the handle toward the tip until a lateral displacement of 1 mm was achieved. The maximum buckling load was recorded in Newtons (N).

The cutting ability test was conducted using a custom apparatus (Odeme OD 127, Odeme Dental Research, Luzerna, Brazil) that connected an endodontic motor handpiece (AI‐Motor; Woodpecker, Guilin, China) to a 500 N load cell of a universal testing machine (DL‐200 MF; EMIC, São José dos Pinhais, Brazil). This device enables the measurement of axial cutting ability. For the test, each instrument was positioned at the top of a simulated straight canal (size 15, taper 0.02) within a bone block model (PCF 10; Sawbones, Vashon, WA, USA). The test began with a 10‐gf preload applied by the universal testing machine, and the instrument was set at 350 RPM with counterclockwise and clockwise angles of 150° and 30° for CC One Blue and Flash, and 30° and 150° for Platinum V.EU. The instrument advanced 3 mm into the canal, then moved backward 2 mm, advancing 1 mm per cycle, repeated until a total of 10 mm was reached. The maximum force encountered was recorded to assess cutting ability, with higher forces indicating lower cutting efficiency (Silva, Pena‐Bengoa, et al., 2024).

Microhardness testing was performed on the reference instruments using a Vickers hardness tester (Duramin; Struers Inc., Cleveland, OH, USA). The specimens were prepared according to ASTM standards (ASTM International, 2017). Initially, three instruments from each group were embedded in acrylic resin to create stable blocks, ensuring secure positioning during the testing process. To maintain consistency and standardization, a flat surface was precisely prepared at the D8 level of each active blade, using abundant water irrigation to prevent overheating and ensure the integrity of the blade. On this flat surface, five indentations were made on each of the three instruments, resulting in a total of 15 indentations per instrument type. A diamond indenter applied a 100 gf load for 15 s (De‐Deus et al., 2017) on the instruments core, and the results were reported as Vickers Hardness Numbers (HVN) at ×40 magnification.

### Statistical analyses

Data normality was assessed using the Shapiro–Wilk test. For normally distributed data, results were expressed as mean values with standard deviations, while non‐normally distributed data were presented as median values with interquartile ranges. The time to fracture, cutting ability, angle of rotation, buckling strength, and microhardness were compared using One‐Way anova, followed by the Tukey Test for post hoc analysis. To compare the maximum torque to fracture and maximum bending load, the nonparametric Kruskal–Wallis test was applied, with pairwise comparisons conducted using the Student–Newman–Keuls Test. A significance level of 5% was set for all statistical analyses (SPSS v22.0 for Windows; SPSS Inc.).

## RESULTS

### Design

**FIGURE 2:**
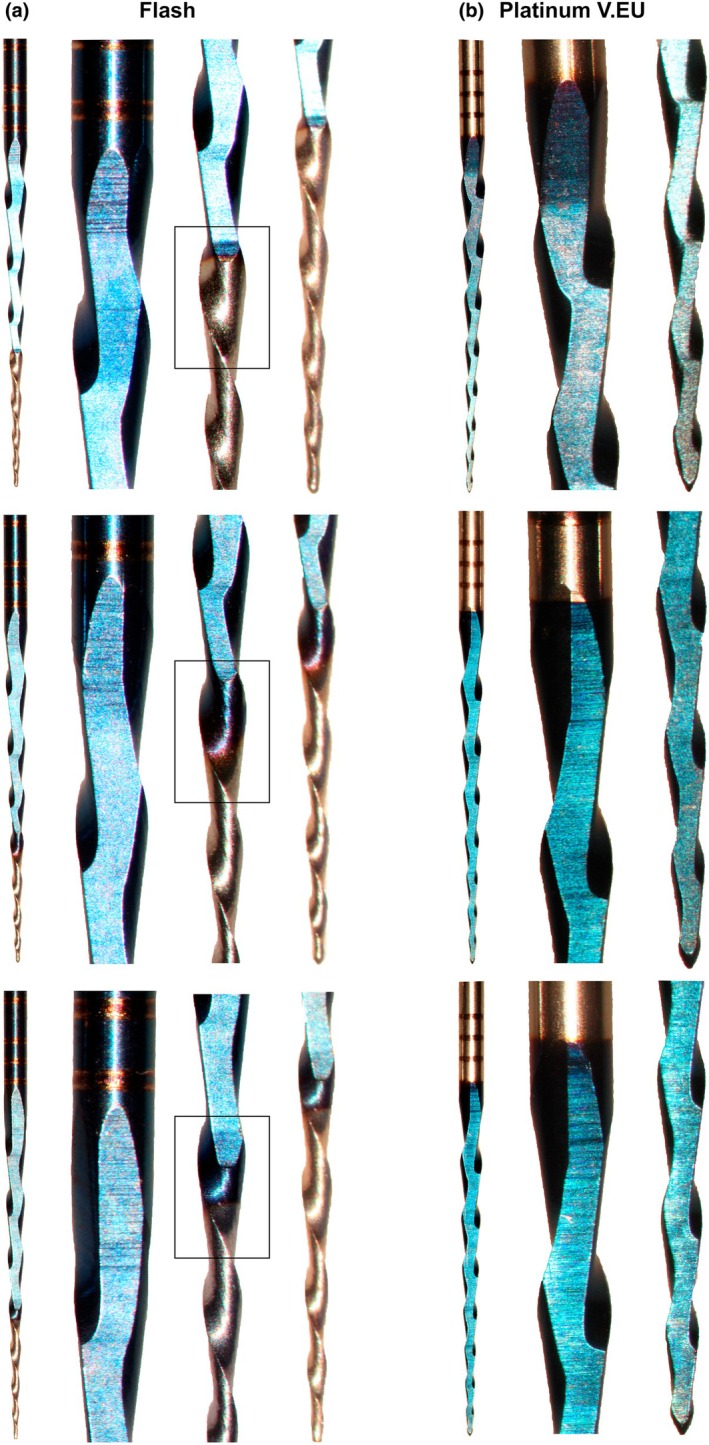
Manufacturing inconsistencies observed in the flat‐side instruments. (a) The Flash instrument exhibited variations in the length of the apical nonflat surface area, with some instruments having this section extending to the beginning of the flat side, while others showed a significantly shorter length. Additionally, the transition between the bluish and white surfaces varied across the instruments, with some displaying a sharp boundary, while others featured a gradual gradient with prominent violet tones (highlighted in rectangles). The nonstandard positioning of the flat side led to discrepancies in blade geometries across the instruments, with these variations being more pronounced in the coronal section but also extending along the entire length of the flat side. (b) Similarly, the Platinum V.EU instruments displayed inconsistencies arising from the nonstandard positioning of the flat side, resulting in significant variations in blade geometries, particularly evident in both the coronal section and the tip areas.

The CC One Blue and Flash instruments measured 18 mm in length, with 8 spirals, 0.44 spirals per millimetre and counterclockwise blade orientation. In contrast, the Platinum V.EU instruments measured 17 mm in length, also had 8 spirals, but featured 0.47 spirals per millimetre and clockwise blade orientation. No significant manufacturing inconsistencies were observed in the nonflat CC One Blue instruments. However, the Platinum V.EU instruments exhibited blade design variations within the same box, mainly due to the nonstandard positioning of the flat side, which affected blade geometry, particularly in the alignment of the cutting edges. The Flash instruments showed similar issues with flat‐side blade geometry irregularities and displayed variations in the length and transition of the nonflat apical area, with some showing abrupt transitions and others exhibiting a gradient with pronounced violet tones (Figure 1). SEM analysis showed that the CC One Blue instrument had a symmetrical blade design, while the Platinum V.EU and Flash instruments had asymmetrical geometries due to the flat side. In the Platinum V.EU instruments, the flat side extended along the entire blade length, while in the Flash instruments, it stopped 6.5 mm short of the tip. None of the instruments had an active tip, and all exhibited typical parallel surface marks from the manufacturing process, which appeared slightly smoothed, with minor irregularities observed among individual instruments (Figure 2).

### Metallurgy

**FIGURE 3:**
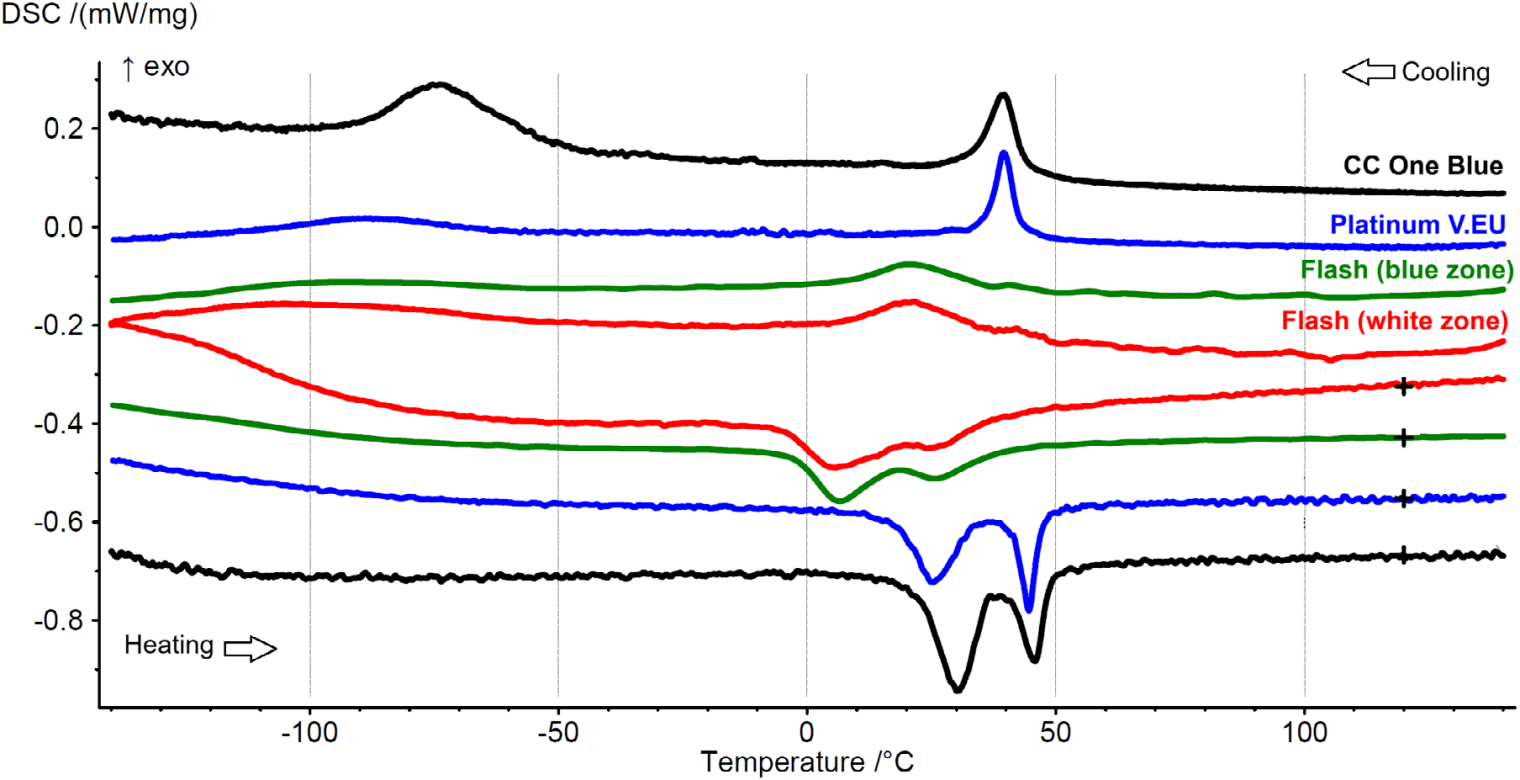
DSC charts illustrating the phase transformation temperatures during cooling (upper lines, reading from right to left) and heating (lower lines, reading from left to right). The analysis of phase transformation temperatures revealed that both the CC One Blue and Platinum V.EU instruments exhibited similar R‐phase transformation temperatures, but differed in the transition from R‐phase to B19' during cooling. The two colour sections of the Flash instruments demonstrated similar transformation temperatures during both cooling and heating. At room temperature (20°C), the Flash instruments displayed a mixed R‐phase and austenitic crystallographic structure, while both the CC One Blue and Platinum V.EU instruments maintained a complete R‐phase structure. Upon reaching body temperature (36°C), the Flash instruments had fully transitioned to the austenitic state, while the CC One Blue and Platinum V.EU instruments retained a mixed R‐phase and austenitic configuration.

All four tested sections confirmed that the instruments were made of a NiTi alloy with an equiatomic ratio of nickel and titanium, without traces of other metals (Table 1). Phase transformation temperature analysis showed that both CC One Blue and Platinum V.EU instruments had similar R‐phase transformation temperatures during cooling and heating, with differences in the transition from R‐phase to B19'. The Flash instruments exhibited similar transformation temperatures across both flat and nonflat sections (Figure 3, Table 1). At room temperature (20°C), Flash instruments displayed a mixed R‐phase and austenitic arrangement, while CC One Blue and Platinum V.EU exhibited a complete R‐phase arrangement. At 36°C, both sections of the Flash instruments fully transitioned to the austenitic state, while CC One Blue and Platinum V.EU remained in a mixed R‐phase and austenitic arrangement (Figure 3, Table 1).

**TABLE 1:** Phase transformation temperatures and elemental composition ratio of tested reference instruments.

Instruments	Section tested	NiTi wire colour	Phase transformation temperatures				Nickel and titanium composition
			Rs	Rf	As	Af	Elements ratio
CC One Blue	Middle	Blue	46.1	30.1	18.9	50.5	1.023
Platinum V.EU	Middle	Blue	45.7	32.4	16.3	50.1	1.010
Flash	Middle	Blue	37.9	9.7	−4.7	40.2	1.022
Flash	Tip	White	36.2	9.5	−4.6	38.6	1.034

Abbreviations: Af, Austenitic finish; As, Austenitic start; Rf, R‐phase finish; Rs, R‐phase start.

### Mechanical performance

Mechanical testing revealed significant differences among the evaluated systems across most parameters (Table 2). The CC One Blue demonstrated superior performance in time to fracture (156 ± 34 s), maximum torque (1.5 N·cm) and buckling strength (372 ± 31 gf) (*p* < .0001). However, no significant differences were observed in the maximum rotation angle (*p* = .602). In terms of flexibility (bending test), the Flash (328 gf) and CC One Blue (341 gf) instruments outperformed the Platinum V.EU (376 gf) (*p* = .006). Also, Flash (121 gf) and CC One Blue (137 gf) outperformed Platinum V.EU (253 gf) in terms of cutting efficiency (*p* < .0001). Conversely, the Platinum V.EU instrument demonstrated higher microhardness (386 ± 45 HVN) compared to both CC One Blue (343 ± 44 HVN) and Flash (359 ± 37 HVN) (*p* = .034).

**TABLE 2:** Mean (standard deviation) or median [interquartile range] results of time to fracture (in seconds), maximum torque (in N.cm), maximum rotation angle (in°), bending load (in gf), buckling load (in gf), cutting efficiency (in gf) and microhardness (in HVN) of tested instruments.

	CC one blue	Platinum V.EU	Flash	*p*‐Value
Time to fracture	156 ± 34^A^	54 ± 18^B^	62 ± 19^B^	<.0001
Maximum torque	1.5 [0.1]^A^	1.1 [0.2]^B^	1.2 [0.1]^B^	.0001
Maximum rotation angle	464 ± 22^A^	415 ± 72^A^	396 ± 43^A^	.602
Bending strength	341 [10]^B^	376 [59]^A^	328 [79]^B^	.006
Buckling strength	372 ± 31^A^	240 ± 23^B^	208 ± 18^C^	<.0001
Cutting efficiency	137 ± 12^B^	253 ± 17^A^	121 ± 12^C^	<.0001
Microhardness	343 ± 44^B^	386 ± 45^A^	359 ± 37^A,B^	.034

*Note*: Different superscript letters in the same line represent statistically significant differences.

## DISCUSSION

This study investigated the impact of active blade designs (nonflat, flat and hybrid) on the mechanical performance of NiTi endodontic instruments. The present findings are particularly innovative, offering a comprehensive comparison of instruments with distinct blade geometries, all manufactured by the same company and tested under consistent conditions. The results demonstrated significant differences across most mechanical parameters, highlighting the critical role of blade design in performance, leading to the rejection of the null hypothesis.

Contrary to the advantages proclaimed by the manufacturer, the hybrid Flash instrument fell short of its intended purpose of balancing flexibility and cutting efficiency. While it demonstrated statistical similarities to the nonflat (CC One Blue) and/or flat‐side (Platinum V.EU) instruments in parameters such as maximum rotation angle, bending strength (flexibility) and microhardness, its mean values were consistently lower across these categories (Table 2). Furthermore, in all other mechanical tests, this novel hybrid instrument failed to outperform either the nonflat or the flat‐side instruments, raising questions about its overall mechanical effectiveness. The flat‐side Platinum V.EU, on the other hand, demonstrated highest mean values only in terms of microhardness. However, this property was offset by its significantly lower performance in key mechanical parameters, including time to fracture, torsional fatigue strength and flexibility (Table 2). These shortcomings suggest that the flat‐side design compromises the instrument's overall mechanical resilience and adaptability, particularly under the stresses encountered in clinical scenarios such as curved or calcified canals. Indeed, the flat‐side blade geometry appears to disrupt the uniform distribution of mechanical stresses along the instrument, increasing the risk of deformation or fracture (Carvalho et al., 2025; Silva et al., 2023). In contrast, the nonflat CC One Blue instrument outperformed the other tested instruments across several critical mechanical parameters, including time to fracture, maximum torque and buckling strength (Table 2). These results underline the ability of nonflat instruments to withstand high‐stress conditions, making it particularly suitable for demanding clinical scenarios. Additionally, its flexibility, as measured by the bending test, was comparable to the hybrid Flash instrument, further highlighting its balanced performance. The superior performance of the CC One Blue highlights the mechanical benefits of the nonflat blade design, which seems to promote a more uniform distribution of mechanical stresses along the instrument, enhancing its structural integrity.

The findings from mechanical tests of flat‐side instruments are more complex to explain and can be attributed to several factors. A closer examination of the phase transformation temperatures of the alloys used to manufacture the tested instruments offers valuable insights. Variations in these transformation temperatures can influence the instrument's behaviour under stress, potentially making it more susceptible to deformation or fracture in certain conditions. In this study, a critical relation was observed between the phase transformation temperatures and the mechanical performance of the instruments. DSC results revealed that the Flash instruments displayed a mixed R‐phase and austenitic arrangement at the mechanical testing temperature (20°C), whereas the CC One Blue and Platinum V.EU instruments exhibited a complete R‐phase. At body temperature (36°C), the Flash instruments transitioned fully to the austenitic state, while the other two instruments retained a mixed R‐phase and austenitic crystallographic arrangement. The earlier transition to the austenitic phase in the Flash instruments may explain their reduced flexibility and strength under mechanical stress, as the austenitic phase is associated with a stiffer, less flexible material state (Thompson, 2000; Zupanc et al., 2018). In contrast, the CC One Blue and Platinum V.EU instruments' retention of the R‐phase contributed to a higher flexibility and superior performance in cyclic fatigue. This characteristic, however, was compromised in the Platinum V.EU instruments due to abrupt changes in blade geometry and lack of standardization on the flat‐side position, potentially exacerbating stress concentrations.

Another explanation for the mechanical behaviour of flat‐side (Platinum V.EU) and hybrid (Flash) instruments in critical tests is provided by the visual analysis of their blades, which revealed manufacturing inconsistencies at the transitions between flat and nonflat sections. These inconsistencies can disrupt the even distribution of mechanical stresses along the instrument, weakening its overall structural integrity. The machining process required to create flat sides generates localized heat, which may disrupt the uniformity of the thermal treatment (Martins et al., 2023). This localized heating can lead to microstructural variations, such as differing phase compositions or residual stresses, forming “thermal islands” within the NiTi matrix (Martins et al., 2023). These inconsistencies potentially weaken the instrument by promoting uneven crack propagation under cyclic fatigue or torsional loading. The transition zones between flat and nonflat sections of the instrument serve as critical stress concentrators due to the abrupt changes in geometry and the potential for metallurgical inconsistencies. These sharp transitions disrupt the uniform distribution of mechanical stresses, leading to localized areas of high‐stress concentration. As a result, these zones are highly susceptible to crack initiation, especially under conditions of cyclic or torsional loading. Once cracks initiate, they are likely to propagate more easily through these weak points, significantly compromising the instrument's structural integrity. This increased vulnerability can lead to premature failure of the instrument, undermining its mechanical performance, particularly in demanding clinical situations where durability and resistance to fracture are important. Studies suggest that variations in heat treatment and geometric design directly affect fracture resistance, making flat‐side instruments more vulnerable to premature failure (Martins et al., 2023; Silva et al., 2023). These findings underscore the complex interplay between design geometry, manufacturing processes and material properties and likely explain the consistently poor performance of flat‐side designs in critical mechanical parameters such as cyclic fatigue resistance and torsional strength.

A distinctive feature of the Flash instruments, aside from their hybrid geometric design, is the dual coloration of the instrument blade. The flat‐side area exhibits a bluish colour, while the nonflat area at the tip appears white. The manufacturer attributes this to a combination of a “double NiTi treatment” and a “no blue nano coating” area (Bondent, 2025). However, this claim is somewhat contradicted by the findings of the present study, as both the white and bluish zones demonstrated identical phase transformation temperatures and similar DSC charts (Figure 3). For heat‐treated NiTi alloys, the grain size of the surface oxide layer typically ranges from 10 to 100 nanometres, depending on the specific thermal treatment applied. While variations in NiTi treatment could potentially influence grain size, the lack of a blue nano coating in the Flash instruments may not be attributable to a double NiTi treatment, as the phase transformation temperatures and DSC charts for both the white and bluish zones were identical. This suggests that the observed coloration difference may result from alternative processes, such as electrochemical treatments, which could alter the surface properties without significantly impacting the phase transformation characteristics. These methods may produce distinct visual effects or surface modifications while maintaining similar mechanical and thermal properties across the different areas of the instrument (Harshini Sai & Hegde, 2023). Unfortunately, the manufacturer does not provide further details about the process, and it remains unclear what impact this feature might have on mechanical performance outcomes. Since the phase transformation temperatures are similar, any potential differences might instead manifest in secondary properties for this type of endodontic instruments, such as corrosion resistance (Harshini Sai & Hegde, 2023) or biocompatibility (Sevost'yanov et al., 2018).

Previous studies testing flat‐side instruments have reported varying degrees of deformation in their active blades, ranging from slight to severe, after use in preparing root canals of extracted teeth (Silva et al., 2023) or simulated canals (Carvalho et al., 2025). These deformations were attributed to the reduced metal mass and the type of thermal treatment applied to the instruments. Furthermore, it has been consistently demonstrated that flat‐side instruments exhibit reduced mechanical resistance compared to their nonflat counterparts (Martins et al., 2023; Silva et al., 2023). As observed in the present study, the limited mechanical performance of flat‐side instruments has been attributed to several critical factors. Inconsistencies in the heat treatment process, such as variations in hardness within the same structure, defects present on the flat surface and alterations in metallurgical properties caused by the grinding process required to create the flat design, play a significant role. Additionally, the reduced metal mass inherent to the flat‐side geometry further compromises the instrument's structural integrity. These combined factors not only diminish mechanical resistance but also significantly increase the risk of premature fracture during their use, highlighting the inherent challenges and limitations associated with the design and manufacturing of flat‐side instruments. Taken together, these findings suggest that, despite the theoretical benefits proposed by the manufacturers (Bondent, 2025; Essential Dental Systems, 2015; Fanta Dental, 2023), the production process for flat‐side instruments faces unresolved challenges, underscoring the need for further refinement in the design and manufacturing of flat‐side and hybrid instruments to achieve the desired mechanical reliability.

One could argue that Platinum V.EU instruments, designed for clockwise rotation, should have been tested under their intended kinematics. While this is a valid consideration, incorporating different kinematic motions in the study would have introduced an additional variable, making it difficult to isolate the influence of blade design on some mechanical properties such as cutting efficiency and cyclic fatigue resistance (Gambarini et al., 2012; Martins et al., 2024). Rather than assessing kinematics as a variable, this study aimed to compare instrument designs under standardized conditions. To ensure a controlled and unbiased comparison, all instruments were tested in reciprocating motion, with necessary adjustments to the larger and smaller angles to accommodate design differences. This approach ensures a methodologically sound evaluation of design characteristics while minimizing confounding factors, allowing for a precise assessment of the effects of blade geometry and metallurgical properties on instrument performance through consistent motion across all instruments.

One of the most debated aspects of evaluating the mechanical behaviour of NiTi instruments in endodontics is the cyclic fatigue resistance test, with both static and dynamic approaches presented in the literature. The static cyclic fatigue test has long been the most widely used method to assess the fracture resistance of NiTi endodontic instruments, while the more recently proposed dynamic test is argued to provide a more clinically relevant simulation of endodontic instrumentation. However, like the static method, dynamic cyclic fatigue tests have several limitations that must be carefully considered when interpreting the results (Hülsmann et al., 2019; Martins et al., 2022; Pedullà et al., 2018; Topçuoğlu et al., 2018). One key issue is the inconsistent contact and load variability, as the instrument undergoes axial or pecking movements, leading to variations in how it contacts the artificial canal. This results in less controlled and reproducible loading conditions. Additionally, the axial movement in dynamic tests intermittently relieves stress on the instrument, potentially extending its fatigue life compared to static conditions, where continuous engagement occurs. This variation makes standardization and reproducibility more challenging. While dynamic testing aims to mimic clinical scenarios, it still does not fully replicate the complex interactions present in real root canal anatomy, such as irregular canal shapes or varying dentin hardness, which influence instrument fatigue. Consequently, dynamic cyclic fatigue tests may overestimate the fatigue resistance of NiTi instruments, as they fail to reflect the continuous stress and localized failure conditions that occur during clinical use. In the present study, the static cyclic fatigue test was chosen because it enables NiTi instruments to be evaluated under identical and controlled conditions, ensuring consistency across all test samples. As a result, the clinical relevance of the testing conditions becomes secondary, since the primary objective is to establish a reliable and valid comparison of the instruments' fatigue resistance.

Heat‐treated NiTi rotary instruments can exhibit different cyclic fatigue behaviour at room versus body temperature. In this study, DSC analysis showed that Flash instruments transitioned from a mixed R‐phase/austenitic state to a fully austenitic phase at 36°C, suggesting that fatigue results at room temperature may not accurately reflect clinical performance (Silva et al., 2024b). This transition likely reduces flexibility and increases stiffness, lowering cyclic fatigue resistance at body temperature. However, testing solely at 36°C is not necessarily more clinically relevant, as temperature fluctuations in the root canal are dynamic rather than static, influenced by irrigation temperature, exposure duration and instrument motion (Atmeh et al., 2024; de Hemptinne et al., 2015; Silva et al., 2025). Thus, applying a constant temperature in laboratory testing may not fully replicate in vivo conditions and could lead to misinterpretations. More importantly, rather than relying solely on temperature‐controlled fatigue testing, combining mechanical tests with DSC analysis provides a more predictive approach, as DSC allows for accurate determination of phase transformation behaviour at different temperatures. This enables a better understanding of how an instrument's mechanical performance is expected to change in clinical conditions, ensuring a more scientifically robust evaluation of NiTi instruments.

A limitation of the current study was the absence of an evaluation of shaping ability, a key parameter for assessing the clinical performance of NiTi instruments. Nonetheless, prior research (Silva et al., 2023) found no significant differences in shaping ability between flat‐side and nonflat instruments. This outcome was attributed to the fact that, although the metallic mass of flat‐side instruments was reduced through the flattening process, their cross‐sectional diameter remained largely unchanged. Consequently, the rotary motion of flat‐side instruments allowed them to create round preparations with dimensions comparable to those of nonflat instruments. Based on this evidence, it is unlikely that the hybrid Flash instrument would perform differently from a nonflat instrument with a similar tip size and taper when tested in anatomically paired root canals. Another limitation of this study is the limited number of assessments used to determine qualitative data, despite efforts to enhance reliability through confirmatory analyses. Additionally, the in vitro nature of this study, while allowing precise control over variables and ensuring reproducibility, does not fully replicate the complexities of clinical conditions. In a clinical setting, factors such as root canal anatomy, operator variability, dentin properties, irrigation protocols and dynamic temperature fluctuations may influence instrument performance. Although differential scanning calorimetry (DSC) provided insights into phase transformation temperatures at body temperature, in vivo conditions introduce variables that cannot be fully simulated in a laboratory environment. These limitations underscore the need for future clinical studies to evaluate the real‐world performance of these instruments under actual endodontic conditions. One advantage of this study was the focus on instruments from a single manufacturer, which helped minimize commercial bias, improve internal validity and provide meaningful insights into how blade design and metallurgy influence mechanical performance. In addition, although the manufacturer did not produce a nonflat NiTi instrument with the exact taper as the flat‐side and hybrid instruments, all tested instruments were heat‐treated and matched in tip size and original S‐shaped cross‐sectional geometry, effectively controlling for potential confounding factors that could influence performance. Another important aspect that deserves attention is the assessment of axial cutting efficiency, which more closely reflects clinical conditions (Silva, Pena‐Bengoa, et al., 2024). Unlike lateral cutting tests—based on the depth of a groove created by sideways movement—axial cutting simulates the actual operative motion of endodontic instruments, which are advanced primarily along the canal's long axis during clinical use.

A key strength of this research lies in its comprehensive multimethod approach, which integrated various analytical techniques to offer a thorough evaluation of instruments' behaviour. By combining metallurgical analysis, mechanical testing and detailed design assessments, the study effectively captured a wide spectrum of performance metrics (Silva et al., 2024a). This approach not only allowed for a deeper understanding of the instruments' structural and functional properties but also facilitated a more accurate interpretation of how different design features influenced their overall performance. Consequently, the present results offer valuable insights for both clinicians and manufacturers. For clinicians, the present findings provide a deeper understanding of how design and metallurgical properties influence the mechanical performance of instruments, guiding informed choices in clinical practice, especially when selecting instruments for challenging cases. For manufacturers, the study highlights key areas for improvement, particularly in optimizing design features and refining production processes to enhance instrument durability, flexibility and cutting efficiency. Ultimately, this research supports the ongoing advancement of endodontic instrumentation, with the aim that future developments will address the growing demands of clinical practice and contribute to improved treatment outcomes.

Based on the current findings, clinicians are strongly advised to exercise caution when utilizing newly designed flat‐side or hybrid instruments, especially in curved or calcified canals. These instruments have demonstrated a tendency to impose increased stress on canal walls during use (Carvalho et al., 2025), exhibit significant distortion under operational conditions (Carvalho et al., 2025; Silva et al., 2023) and display limited mechanical performance (Martins et al., 2023; Silva et al., 2023). Taken together, these factors substantially heighten the risk of instrument fracture during clinical procedures, emphasizing the need for careful case selection and application. This recommendation aligns with the findings of Jeong et al. (2024), who reported that the side flattening of endodontic NiTi instruments could significantly reduce their mechanical resistance. The authors emphasized the need for careful handling of such instruments to minimize potential complications during clinical use.

## CONCLUSIONS

This study provides a comprehensive evaluation of the influence of blade design and metallurgical composition on the mechanical performance of NiTi endodontic instruments. The findings reveal that instruments with flat‐side (Platinum V.EU) and hybrid (Flash) blade designs demonstrated inferior mechanical properties compared to the conventional nonflat instrument (CC One Blue). Specifically, the CC One Blue instrument exhibited superior performance in terms of time to fracture, maximum torque and buckling resistance, while maintaining flexibility and cutting efficiency. In contrast, the flat‐side Platinum V.EU showed higher microhardness but compromised cyclic fatigue resistance and flexibility. The hybrid Flash instrument, which was designed to combine the benefits of both blade geometries, did not outperform either of the other designs in most mechanical tests, raising concerns regarding its clinical reliability and performance.

## AUTHOR CONTRIBUTIONS

Emmanuel J. N. L. Silva: conceptualization, analysis, experimental procedures, writing, review and editing (lead). Jorge N. R. Martins: conceptualization, analysis, experimental procedures, writing, review and editing (lead). Thyago Oliveira Cardoso: experimental procedures, writing. Mylena do Rosário Pereira: experimental procedures. Murilo Priori Alcalde: experimental procedures, writing. Victor T. L. Vieira: experimental procedures, writing. Abayomi O. Baruwa: experimental procedures. Francisco Manuel Braz Ferndandes: experimental procedures. Marco A. Versiani: conceptualization, analysis, experimental procedures, writing, review and editing (lead).

## FUNDING INFORMATION

This study was partially funded by FAPERJ and CNPq.

## CONFLICT OF INTEREST STATEMENT

The authors declare that they have no competing interests with regard to this paper.

## ETHICS STATEMENT

This laboratory study did not involve human participants or animal subjects; therefore, ethical approval was not required.

## Supporting information


Figure S1

## Data Availability

Data available on request from the authors.
